# Post-market surveillance for literature on QuantiFERON TB Gold Plus: what improvements can artificial intelligence bring?

**DOI:** 10.5588/ijtldopen.25.0711

**Published:** 2026-05-11

**Authors:** J. Reniewicz, R. Alagna, L. Kordylas, M. Latacz, V. Suryaprakash, U. Nowak, A. Kois-Ostrowska, J. Weleszczuk, A. Blacha, V. Nikolayevskyy

**Affiliations:** 1 QIAGEN Wroclaw Sp. z o.o., Wroclaw, Poland;; 2 QIAGEN S.p.A., Milan, Italy;; 3 QIAGEN Hilden GmbH, Hilden, Germany;; 4 QIAGEN Manchester Ltd, Manchester, UK;; 5 Imperial College London, London, UK.

**Keywords:** tuberculosis, diagnosis, interferon-gamma release assay, in vitro diagnostic medical device, natural language processing, literature searches

## Abstract

**BACKGROUND:**

Post-market surveillance (PMS) under the European Union In Vitro Diagnostic Regulation (IVDR) demands proactive, literature-based evidence, but mature assays like QuantiFERON TB Gold Plus (QFT-Plus) generate volumes of peer-reviewed and other literature that can strain manual workflows.

**METHODS:**

We ran a comparative study of an AI-enabled literature-surveillance platform (jointly developed with Huma.ai called the Huma.ai Platform) versus manual search for QFT-Plus PMS. PubMed and PubMed Central were queried for publications in 2024; human studies published in English underwent duplicate screening and full-text appraisal. Outcomes were yield, precision, overlap/unique entries, and reviewer time.

**RESULTS:**

The Huma.ai Platform retrieved 673 records, with 661 relevant to screening (98.21% precision). Manual searching retrieved 111, with 106 relevant to screening (95.50% precision): there were 103 shared and three manual-only items (metadata gaps). The Huma.ai Platform contributed 561 unique papers, 5 of which were excluded after full-text appraisal. In total, 664 articles were evaluated; no new safety signals were identified. Screening time averaged ∼16 s per article with Huma.ai Platform versus ∼60 s manually; full-text time (∼15 min per article) was similar.

**CONCLUSION:**

AI-assisted surveillance substantially increases coverage and reduces screening effort while maintaining high precision. Thus it supports efficient, reproducible PMS for QFT-Plus.

Post-market surveillance (PMS) is a critical aspect for ensuring patient health and safety after a medical device (including in vitro diagnostic [IVD] assays) has been launched on the market. In line with WHO guidance, PMS should integrate multiple evidence streams – scientific literature, complaint/vigilance data, registries, real-world performance studies, and feedback from users – to detect emerging risks, and drive corrective and preventive actions.^1^ With the introduction of the In Vitro Diagnostic Regulation (IVDR) in the European Union (EU), the requirements for PMS have significantly increased, leading to a higher workload and associated costs for manufacturers. Accurate data searches and timely documentation updates are critical to maintaining the availability of assays on the market, as they help identify and mitigate potential risks.

The QuantiFERON TB Gold Plus (QFT-Plus, QIAGEN, GmbH) is a widely used IVD assay supported by a large and continuously growing body of publications and clinical recommendations.^2–5^ The breadth of evidence, together with evolving requirements under the IVDR, creates unique PMS challenges mainly associated with resources needed to ensure comprehensive literature surveillance, prompt detection of safety and performance signals, and timely maintenance of technical documentation. Conventional manual workflows are resource-intensive, difficult to scale, and susceptible to delays and inconsistency. Artificial intelligence (AI) offers a scalable approach to PMS. AI, particularly machine learning (ML) and natural language processing (NLP), can be used to automate literature surveillance, broaden coverage, accelerate and sharpen signal detection, and reduce reviewer time and cost.^6^ These capabilities are especially valuable for mature assays with an extensive evidence base, such as QFT-Plus, enabling time- and cost-effective proactive refinement of product performance, sustained safety and effectiveness, competitive advantage, and a reduced risk of market withdrawal. Beyond PMS, the same methods can help support diagnosis, clinical decision-making, drug discovery, and risk management.^7,8^

To facilitate efficient adherence to EU IVDR, QIAGEN and Huma.ai co-developed a post-market intelligence platform (Huma.ai Platform) that systematises risk and performance evaluation based on literature surveillance. The platform continuously searches and classifies publications aligned to a device’s intended use and, for pathogen-based IVDs, to genetic regions of interest, using context-aware algorithms to capture evidence on target genetic variability. The resulting workflows streamline key PMS deliverables (e.g., Post-Market Surveillance Reports [PMSR], Periodic Safety Update Reports [PSUR], or Post-Market Performance Follow-Up [PMPF] Evaluation Reports) while improving traceability, reproducibility, and overall efficiency.^6^ In this study, using QFT-Plus as an exemplar of a well-established mature diagnostic assay with a global footprint, we assess whether the platform improves PMS performance versus conventional manual methods by increasing recall and precision of literature-based signal detection, reducing time-to-evidence and reviewer effort for PMS outputs, and enhancing the traceability, reproducibility, and decision utility of the compiled documentation.

## METHODS

We conducted a comparative methods study to evaluate an AI-enabled literature-surveillance workflow (Huma.ai Platform) versus conventional manual searches for PMS evidence on QFT-Plus. The Huma.ai Platform is a bespoke tool which was developed in a collaboration between QIAGEN and Huma.ai. The platform uses approaches from classical NLP (including pattern recognition, but excluding generative AI or large language models) to find relevant publications; this not only ensures process transparency but also generates reproducible and deterministic results.^6^ Searches were limited to items e-published in 2024, and run on PubMed and PubMed Central (PMC). The AI workflow and comparator were modelled on a previously described approach for PMS literature retrieval and assessment established at QIAGEN.^6^

Articles were eligible for inclusion if: i) full text was available in English; ii) the study was conducted in humans; and iii) the full text contained information pertinent to device safety and/or performance within the intended use of QFT-Plus (e.g., accuracy, reliability, analytical/clinical performance, and risk signals). These criteria mirror literature methods for IVDs established as a part of PMS procedures at QIAGEN.

The AI-based search strategy utilised a validated, multi-algorithmic method that is not transferable to linear searches, as it cannot be replicated in most standard user interface search engines. The manual search query, constructed using Boolean operators, is as follows: (‘quantiferon’ [All fields] OR ‘QFT’ [All fields]) AND (‘mycobacterium tuberculosis’ [All fields] OR ‘mycobacterium’ [All fields] OR ‘tuberculosis’ [All fields] OR ‘MTB’ [All fields] OR ‘M. tuberculosis’ [All fields] OR ‘TB’ [All fields] OR ‘latent tuberculosis’ [All fields] OR ‘latent tb infection’ [All fields] OR ‘LTBI’ [All fields] OR ‘tuberculosis’ [MeSH Terms] OR ‘Latent Tuberculosis’ [MeSH Terms]).

Screening and full-text review of all identified articles (i.e., by manual and AI-based search) were performed by a total of five reviewers. To ensure data reliability and validity, a quality control (QC) assessment was performed by an independent reviewer who was not involved in the initial screening process. Articles for QC were selected using a random sampling method to ensure that each article had an equal probability of selection. The sample size was calculated using the formula: the square root of the total number of evaluated articles plus 1, and the selection was performed in Microsoft Excel using the RANDBETWEEN function. Of the data analysed, 12 articles were selected for QC for manual approach, and 27 for AI-based search. Any disagreements between reviewers were resolved by a third reviewer. For articles meeting inclusion criteria, we extracted whether the report contained safety and/or performance information relevant to QFT-Plus and its intended use. Procedures were consistent with prior PMS studies comparing manual versus AI-assisted retrieval.

The primary outcomes were the number of relevant (meeting all the inclusion criteria listed above) articles identified by each approach and the precision, defined as relevant articles divided by total articles screened for that approach (for both manual keyword search and Huma.ai Platform output). Secondary outcomes included reviewer-reported time for screening and full-text assessment for each approach.

We performed a descriptive analysis comparing effectiveness of manual process versus the AI-assisted one. For each approach, we calculated retrieval yield (total records) and precision (relevant/retrieved). We quantified set overlap (identified by both methods) and method-unique records (AI-only, manual-only). AI-only records underwent full-text verification by a team of five reviewers, and we recorded the small fraction excluded at full text despite initial relevance at screening. Manual-only records were traced to indexing/availability issues (e.g., missing abstracts/links) and reviewed to confirm whether their inclusion would alter risk conclusions. Across all relevant 2024 articles (overlap + AI-unique), we assessed utility for PMS by determining whether any report introduced new or previously unrecognised safety/performance risks; none were found. Reviewer effort was summarised as mean screening time per record (title/abstract/Region of Interest [ROI]) and mean full-text appraisal time, and screening efficiency was expressed as the fold-reduction versus manual review, with full-text review times by experts noted as comparable across methods.

## RESULTS

The articles identified in 2024 by both Huma.ai Platform and manual searches were evaluated to compare the methodologies based on the number of results obtained and their quality and utility in PMS literature assessment. The summary of the results is provided in the Table. A comparative analysis of the initial screening results between the Huma.ai Platform and the manual review process revealed that the Huma.ai Platform achieved a slightly higher precision rate compared to the manual approach. Additionally, the AI-assisted method identified a substantially greater number of articles.

**Table.** The comparison of the manual and AI-assisted search methods.

Parameter	Manual method	Huma.ai Platform
Total articles identified	111	673
Relevant articles^A^	106	661
Precision rate (%)^B^	95.50%	98.21%
Method-unique articles^C^	3	561
Average abstract screening time per article	∼60 s	∼16 s
Average full-text review time	∼15 min	∼15 min

A Relevant articles were defined as those that explicitly reference the evaluated QFT-Plus.

B Precision rate represents the proportion of articles classified as relevant (i.e., that met all inclusion criteria) relative to the total number of articles identified by either manual method or the Huma.ai Platform.

C Method-unique articles refer to those articles that were missed by one search method but successfully identified by the alternative approach.

A total of 103 articles were identified by both methods. Huma.ai Platform did not retrieve three articles found by the manual assessment. Further investigation showed that these were not available to the Huma.ai Platform due to incorrect PubMed citation (i.e., unavailable abstract and lack of full text hyperlink). These articles were analysed manually outside the PubMed, and none were associated with any unidentified product risk. Despite similar precision values between the two methods, 561 findings were unique to the Huma.ai Platform and were not retrieved in the manual procedure. All relevant 664 articles (561 unique to the Huma.ai Platform and 103 identified by both methods) were evaluated for potential information on new and unknown risks associated with the product's use in the market, as well as for any safety-related data concerning the product use. Full-text assessment of those articles showed that five – although marked as relevant during screening – did not meet the inclusion criteria for PMS literature assessment. None of the remaining relevant articles collected during 2024 resulted in a new risk identification.

The time needed to perform the literature review and screen the results was significantly reduced with the Huma.ai Platform compared to the manual methodology. The average time needed to confirm the relevance based on the title, abstract, and ROI was 16 s/article on average, whereas manual screening required approximately 1 min per article. The duration required for full-text analysis by an expert remained consistent across all used methods, averaging approximately 15 min per article.

## DISCUSSION

This study evaluated the performance of the Huma.ai Platform in the context of the PMS of the QFT-Plus assay, comparing it to traditional manual literature review methods. The findings demonstrate that AI-assisted surveillance offers substantial advantages in terms of efficiency, consistency, and comprehensiveness, while maintaining high precision in identifying relevant literature.

In 2024, Huma.ai identified 673 articles, of which 661 were deemed relevant after initial screening – yielding a precision rate of 98.21%. The manual method retrieved 111 articles, with a slightly lower precision of 95.50%. Notably, 103 articles were identified by both methods, and only three articles were missed by the Huma.ai Platform due to metadata issues in PubMed. These omissions were not associated with any new or unidentified product risks. Importantly, 561 articles were uniquely identified by the Huma.ai Platform, of which five did not meet the inclusion criteria after full-text review by an expert.

The time advantage provided by the Huma.ai Platform was marked: relevance screening approximately 16 s per article versus approximately 60 s manually, while full-text analysis appraisal times for an expert were comparable across both approaches. These findings are consistent with prior studies demonstrating that AI can accelerate literature triage without compromising screening precision.^9–11^

Automated systems like the Huma.ai Platform apply standardised and well-calibrated algorithms across monitoring cycles, reducing inter-reviewer variability, and enhancing consistency and reproducibility – attributes that are especially valuable in regulated settings emphasising traceability and auditability.^6,12,13^ The AI system’s ability to maintain stable performance over time supports its integration into routine PMS workflows, especially under the IVDR framework.^14^

Although no new risks were identified, the breadth of literature captured by the Huma.ai Platform provides reassurance that the surveillance process was comprehensive and systematic, aligning with WHO’s Good Vigilance Practices that underscore the importance of ongoing literature monitoring – even when no emerging signals are detected.^15^ Such diligence, particularly for well-established products like QFT-Plus where the risk profile is already well-documented,^5,16^ strengthens confidence among regulators, thousands of health care professionals, and many thousands of patients tested annually.

Manual literature reviews rely heavily on subjective keyword selection and MeSH term interpretation, potentially introducing variability and bias. In contrast, AI systems leverage NLP and ML to identify semantically relevant content, reducing reliance on rigid indexing.^10,17^ Nevertheless, algorithmic term selection must be validated to ensure transparency and avoid overfitting or bias.^12,18^ The capacity of AI systems to process large volumes of literature can widen surveillance scope and, in principle, improve sensitivity to rare or emerging signals. Studies have shown that AI-enhanced pharmacovigilance systems can identify adverse events earlier and more sensitively than traditional methods.^19,20^ However, increasing the number of results does not guarantee better signal detection; the value derives from precision, prioritisation (e.g., ROI tagging), and expert adjudication. As highlighted by Farah et al.,^21^ the quality and interpretability of AI-generated evidence remain critical. Hence, expanded retrieval should be coupled with robust validation and human oversight to ensure that downstream PMS decisions are meaningful and proportionate.

From a regulatory-ethics perspective, deploying AI within PMS should align with expectations of transparency, human oversight, risk management, and lifecycle monitoring under IVDR/MDR and related policy discussion.^13,14^ Similarly, the FDA’s AI/ML Action Plan highlights explainability, real-world performance monitoring, and lifecycle management.^21^ Practical enablers include auditable workflows, documented reviewer decisions, and software version control, recognising that this study used literature sources indexed in PubMed/PMC.

This evaluation has some limitations. Scope was restricted to 2024 e-publications, English full text, and PubMed/PMC indexing; we did not include Embase/Scopus, preprints, grey literature, regulatory reports, or non-English sources, so absolute recall is unknown. No external reference set was used; relevance was judged by multiple reviewers, all arriving at the same conclusions. Huma.ai Platform’s ROI tags may have primed screening, and time measurements were unblinded and vulnerable to learning or order effects. Retrieval depended on metadata quality – three manual-only records reflected incomplete PubMed citations – and we assessed a single software configuration over one cycle, so updates or re-indexing could alter results. The test case (QFT-Plus) is a mature assay, which may limit generalisability to devices with sparse or rapidly evolving evidence; outcomes focused on retrieval and screening effort rather than downstream PMS deliverables. Finally, we assessed publications only and did not integrate other PMS evidence sources (e.g., complaints/vigilance, registries, real-world performance, or user feedback); therefore, absence of signals in the literature may not reflect the broader post-market risk landscape beyond PMS, and structured outputs from the Huma.ai Platform can support R&D, regulatory submissions, and health-economic analyses. AI tools like LiteRev^9^ and BioBERT^22^ demonstrate how literature-derived insights can inform drug repurposing, biomarker discovery, and clinical trial design. Integrating AI outputs with real-world data^23^ and patient-reported outcomes could further enhance post-market evidence generation. Future work should explore hybrid human–AI workflows that combine the scalability of automation with the contextual judgement of experts, consistent with regulatory guidance emphasising explainability, traceability, and human oversight.^13,14^

## CONCLUSION

This study confirms that AI tools like the Huma.ai Platform can significantly enhance the efficiency, consistency, and comprehensiveness of PMS for mature IVDs such as QFT-Plus. While no new safety signals were identified, the system’s ability to process large volumes of literature with high precision and speed supports its role in modern pharmacovigilance. As regulatory frameworks evolve, the integration of explainable, validated AI systems will be essential to ensure ethical, transparent, and effective surveillance.
